# Prevalence and Virulence Determinants of *Staphylococcus aureus* in Wholesale and Retail Pork in Wuhan, Central China

**DOI:** 10.3390/foods11244114

**Published:** 2022-12-19

**Authors:** Zhihao Zhu, Xiaoying Liu, Xingyu Chen, Geng Zou, Qi Huang, Xianrong Meng, Xiaoying Pei, Zhou Chen, Rui Zhou, Dongliang Hu, Mei Liu, Shaowen Li

**Affiliations:** 1 Key Laboratory of Preventive Veterinary Medicine in Hubei Province, College of Veterinary Medicine, Huazhong Agricultural University, Wuhan 430070, China; 2 Wuhan Agricultural Comprehensive Law Enforcement Inspector Corps, Wuhan 430010, China; 3 Department of Zoonoses, Kitasato University School of Veterinary Medicine, Towada 034-8628, Japan

**Keywords:** antimicrobial resistance, enterotoxin, food safety, MRSA, pork, *Staphylococcus aureus*, virulence factor

## Abstract

*Staphylococcus aureus* is one of the major foodborne pathogens and can cause serious foodborne illness in humans by foods contaminated with *S. aureus* enterotoxins. In recent years, livestock-associated *S. aureus* has been a major public health concern for humans and has emerged in various countries globally. China is one of the largest producers of pigs and pork in the world. However, there are few studies on the detailed genotypic and pathogenic characterization of pork-associated *S. aureus* in China. In this study, the prevalence, antimicrobial resistance, and genotypic characteristics of *S. aureus* in raw pork in Wuhan, China, were investigated through multilocus sequence typing (MLST), staphylococcal protein A gene (*spa*) typing, and whole-genome sequencing analysis. A total of 518 *S. aureus* isolates (16.9%) were isolated from 3067 retail and wholesale pork samples. The prevalence of *S. aureus* in retail pork (22.7%) was significantly higher than in wholesale pork (15.1%), while the proportion of multidrug-resistant (MDR) isolates in wholesale pork (12.9%) was significantly higher than in retail pork (6.2%). Among the isolates, 10.8% were resistant to three or more antibiotics, with higher rates of resistance to penicillin (88.8%) and erythromycin (58.1%). A total of 28 sequence types (STs) were identified in the 518 isolates, and the predominant type was ST7 (57.5%), followed by ST5 (9.1%). In addition, based on the whole-genome sequences of 39 representative strains, 17 *spa* types were identified among the isolates, of which t899, t091, and t437 were the most common. Furthermore, 19 staphylococcal enterotoxin (SE) and SE-like (SEl) toxin genes were detected in the isolates, of which *selw* was the most common type (100%), followed by *sei*, *sem*, *seo*, *seu*, and *selv* (46.2%); *sey* (35.9%); and *sea*, *seg*, and *sen* (33.3%). This study found for the first time that ST7-t091-*selw* and ST9-t899-SCCmecXII-*selw* were the predominant genotypes of *S. aureus* in pork in China, which indicated the spreading of *S. aureus* with multiple virulence factors, especially with new SE/SEl types in pigs and pork, is a serious new challenge for food safety. Good hygiene and good production practices to prevent interspecies transmission and cross-contamination of *S. aureus* in the pig–pork chain are of great significance to public health.

## 1. Introduction

*Staphylococcus aureus* (*S. aureus*) is a major commensal pathogen, which induces various infections in animals and humans [1,2]. *S. aureus*-induced infectious diseases in humans are mainly caused by the strains with capacity to produce multiple virulence factors, such as leukocidins, exfoliative toxins, hemolysins, fibronectin-binding proteins, clumping factors, collagen-binding protein, staphylococcal enterotoxins (SEs), and SE-like toxins [3,4]. In recent years, the frequent use of antimicrobials and high feeding densities on pig farms have facilitated the emergence and spread of *S. aureus* and livestock-associated methicillin-resistant *S. aureus* (LA-MRSA). Antimicrobial-resistant *S. aureus* has been isolated from pigs in farms, slaughterhouses, and markets in many countries [1,2]. The increasing amount of methicillin-resistant *S. aureus* (MRSA) has brought enormous difficulties and serious challenges to medical care and public health. In addition, SE and/or SEl-producing *S. aureus* is also one of the world’s leading causes of food consumption-related disease outbreaks, causing food poisoning worldwide [5,6]. To date, 29 types of SEs and SE-like toxins (SEls) have been identified, including the classic types of SEs (SEA-SEE) and novel SE/SEls (SEG-SElZ, SEl01, and SEl02) [4,7]. Certain new SEs/SEls have been reported as potential causes of foodborne disease outbreaks [8,9]. The genes of SEs and SEls are typically located on mobile genetic elements (MGEs) [4,10,11]. SEs/SE1s and antimicrobial resistance genes can be transferred among *S. aureus* strains by the horizontal transfer of MGEs, which accelerate the evolution of pathogenic *S. aureus* strains among animals and humans, as well as increase the risk of infection and food poisoning in humans [4,12,13]. Investigating and elucidating the epidemiological and genotypic characteristics of *S. aureus* in animals and their products are important for controlling bacterial transmission and disease occurrence in animals and humans.

Epidemiological studies showed that *S. aureus* has often been isolated from pigs, which are a key host for *S. aureus*, especially for LA-MRSA [14,15]. LA-MRSA can be transmitted to humans through occupational contact with pigs, pork, and their products [16,17]. Previous studies have revealed that LA-MRSA strains have different genetic backgrounds and different antibacterial resistance and carry different types of SCC*mec* and virulence factors [18,19]. The most prominent LA-MRSA strains in Europe and the United States belong to sequence type 398 (ST398) [20,21,22], which has also been identified in Australia [23] and New Zealand [24]. In most Asian countries, however, the most prominent genotype belongs to ST9 [19,25,26]. Recently, methicillin-susceptible *S. aureus* (MSSA) ST398 has been recovered from pigs, retail meat, and humans in Germany, France, the Unite States, and China [15,21,27,28,29]. The infections caused by MSSA ST398 have been described in human diseases [20,30]. These studies demonstrate that livestock-associated *S. aureus* isolates, both of MRSA and MSSA, are significant food safety hazards. Handling food-producing animals, especially pigs, and eating pork contaminated with *S. aureus* are potential sources of zoonotic transmission in humans.

China is one of the largest producers of pigs and pork in the world [31,32]. In this study, we investigated the prevalence, antimicrobial resistance, virulence genes, and genotypic characteristics of *S. aureus* including MSSA and MRSA in raw pork in Wuhan, a city of central China. Our study found for the first time that ST7-t091-*selw* was the predominant genotype of MSSA, and ST5-t899-SCC*mec*XII-*selw* was a common genotype of MRSA in pork, suggesting that these two main genotypes of *S. aureus* may have been involved in spread along the pork processing chains.

## 2. Materials and Methods

### 2.1. Bacterial Isolation and Identification from Raw Pork

A total of 3067 pork samples were collected from raw pork markets in Wuhan city, during 2016–2017. Of these, 2353 samples were from different stalls in three wholesale markets, and 714 samples were from nine retail markets. The sampling covered 38 abattoirs supplying raw pork for Wuhan city. About 20 g of meat was taken from each sample and stored in a refrigerator. Isolation of *S. aureus* was performed according to the Chinese National Standard GB4789 (https://www.chinesestandard.net/PDF/English.aspx/GB4789.10-2010 (accessed on 20 January 2016)) with some modifications. The presence of *S. aureus* in suspected positive broths was confirmed using polymerase chain reactions (PCR) to identify the *femB* gene with the primers (*femB*-F, CATGGTTACGAGCATCATGG, and *femB*-R, AACGCCAGAAGCAAGGTTTA) [33,34]. A loopful from the incubated tubes was streaked onto a Baird-Parker agar plate that was supplemented with 5% egg yolk and tellurite and incubated at 37 °C for 40–48 h. The presumptive *S. aureus* colonies were further identified by using an automated microbiological identification system (BD Phoenix ^TM^PMIC/ID-55).

### 2.2. Antimicrobial Resistance Testing

The antimicrobial resistance of *S. aureus* isolates was tested by determining the minimal inhibitory concentrations (MICs) using an automated antibiotic susceptibility analysis system (BD Phoenix TMPMIC/ID-55), and break points from the Clinical and Laboratory Standards Institute (CLSI) were used for MIC interpretation [34] (Table S1). Eight antimicrobial agents of four classes were used in this study, including beta-lactams (penicillin (PEN), oxacillin (OXA), and amoxicillin/clavulanate (AMC)), macrolide-lincosamide-streptogramines (erythromycin (ERY), clindamycin (CLI), and quinupristin/dalfopristin (SYN)), aminoglycosides (gentamicin (GEN)), and folate pathway antagonists (trimethoprim/sulfamethoxazole (SXT)). Antimicrobial resistance (AMR) was defined as resistant to at least one antimicrobial, whereas multidrug resistance (MDR) was defined as resistant to three or more classes of antimicrobials in this study. MRSA was defined as the oxacillin-resistant isolate harboring the *mec*A or *mec*C gene [33,34,35]. *S. aureus* ATCC29213 was used as the quality control strain.

### 2.3. Detection of Methicillin Resistance Genes

The presence of the *mec*A and *mec*C genes in the genomic DNA of *S. aureus* isolates from the pork samples was detected by PCR using primers (*mec*A-F, TGGTATGTGGAAGTTAGATTGGGAT; *mec*A-R, CTAATCTCATATGTGTTCCT GTATTGGC; *mec*C-F, CATTAAAATCAGAGCGAGGC; and *mec*C-R, TGGCTGAA CCCATTTTTGAT) and the conditions as previously reported [33,35,36].

### 2.4. Multilocus Sequence Typing (MLST)

The isolates from pork were recovered in lysogen broth (LB) at 37 °C for 12 h with shaking, and the genomic DNA template was extracted using a heat boiling method. MLST was performed following the protocol described elsewhere [37]. Seven housekeeping genes—carbamate kinase (*arcC*), shikimate dehydrogenase (*aroE*), glycerol kinase (*glpF*), guanylate kinase (*gmk*), phosphate acetyltransferase (*pta*), triosephosphate isomerase (*tpi*), and acetyl coenzyme A acetyltransferase (*yqiL*)—were amplified by PCR. The alleles and sequence types (STs) were identified using the scheme published on the multilocus sequence typing databases (https://pubmlst.org/organisms/staphylococcus-aureus (accessed on 24 September 2018)).

### 2.5. Whole-Genome Sequencing and Data Analysis

Thirty-nine representative *S. aureus* strains were selected for whole-genome sequencing (WGS), including 19 MRSA strains and 20 MSSA strains; 27 strains were from the wholesale markets, and 12 strains were from the retail markets. Library preparing and de novo bacterial sequencing was performed using the Illumina platforms with NovaSeq 6000 Sequencing System (Illumina, San Diego, CA, USA) in Annoroad Gene Technology Co., Ltd, Beijing [38]. The data volume for each bacterial sample was 2 GB, with a sequencing depth of 300×. After quality control and data filter, the clean reads were assembled using SPAdes (v3.14.1) with the “isolate” option, and the default K-mers were 21, 33, 55, and 77. Quast was used to evaluate the quantity of the assembled contigs. The obtained genome contigs were used for subsequent analysis, including screening of resistance and virulence genes using abricate (v1.0.0) (Seemann, Abricate, Github, https://github.com/tseemann/abricate (accessed on 3 January 2021)) with the appropriate database, the comprehensive antibiotic resistance database (CARD, http://arpcard.mcmaster.ca (accessed on 8 November 2021)), and the virulence factor database (VFDB, http://www.mgc.ac.cn/VFs/ (accessed on 8 November 2021)), and the plasmid types were determined using PlasmidFinder software (https://cge.food.dtu.dk/services/PlasmidFinder/ (accessed on 8 November 2021)). The genes recorded in the CARD and VFDB databases were compared as reference genes, and the compared genes were determined to be the genes carried by the strain. The resistance genes (e.g., *arlR*, *arlS*, *mgrA*, *mepA*, *mepR*, *tetK*, *tetL*, *blaZ*, *ANT*, *aph*, *aad*, *lnuA*, *ermB*, *ermC*, *fusB*, and *dfrG*), virulence genes (*icaA*, *isdA*, *lio*, *ssp*, *hlb*, *hld*, *sak*, *scn*, *chp*, *clfA*, *clfB*, *fnbA, fnbB*, *lukF*-PV, *lukS*-PV, *spa*, and *tst*), and enterotoxin genes (*sea*, *seb*, *sec*, *sed*, *see*, *seg*, *sei*, *selj*, *sek*, *sem*, *sen*, *seo*, *selu*, *selv*, *selw*, and *sey*) were identified [39,40]. For the spliced genomic contigs, the core SNP tree was extracted and constructed using Snippy software (v3.2), and the recombination sequences were detected and eliminated using gubbins software (Seemann, Snippy, https://github.com/tseemann/snippy (accessed on 13 July 2021)). The maximum likelihood tree of core SNPs was constructed using RaxML (v8.0) software with the reference genome *S. aureus* NCTC8325 (txid: 93061) and 1000 bootstrap support, and the tree was visualized and annotated in iTOL version 4 [41]. The *S. aureus* isolates were also investigated by *spa* typing using the publicly available Ridom SpaServer (www.spaserver.ridom.de (accessed on 15 July 2021)). The SCC*mec* typing of MRSA was performed by SCC*mec*Finder (https://cge.food.dtu.dk/services/SCCmecFinder/ (accessed on 17 July 2021)).

### 2.6. Statistical Analysis

Data analyses were performed using SPSS software, ver. 26.0 (SPSS Inc., Chicago, IL, USA). The 95% confidence interval (CI) was calculated using the method described by Ross [42]. The statistical significance between the percentages of different groups was compared using the Chi-squared (χ2) test. The *p* value of <0.05 was deemed to be significant.

## 3. Results

### 3.1. Prevalence of S. aureus and MRSA in Wholesale and Retail Pork

A total of 518 *S. aureus* (16.9%) isolates were isolated from 3067 pork samples, which included 356 isolates from the wholesale markets (356/2353, 15.1%) and 162 isolates from the retail markets (162/714, 22.7%). The prevalence of *S. aureus* in retail pork was significantly higher than that in the wholesale pork, and the prevalence of *S. aureus* in retail pork in winter was significantly higher than that in the other three seasons (*p* < 0.05) (Table 1).

### 3.2. Antimicrobial Resistance Profiles

**Figure 1:**
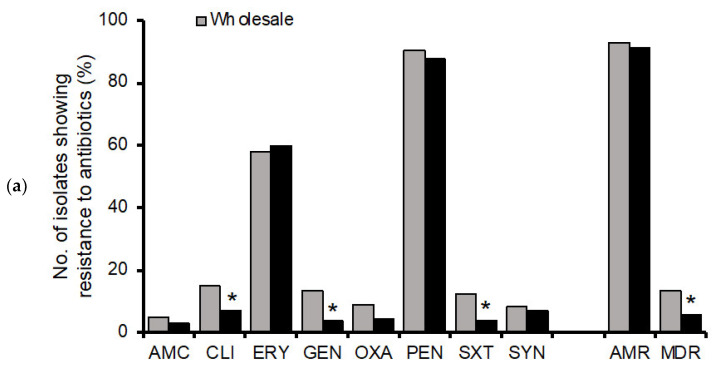

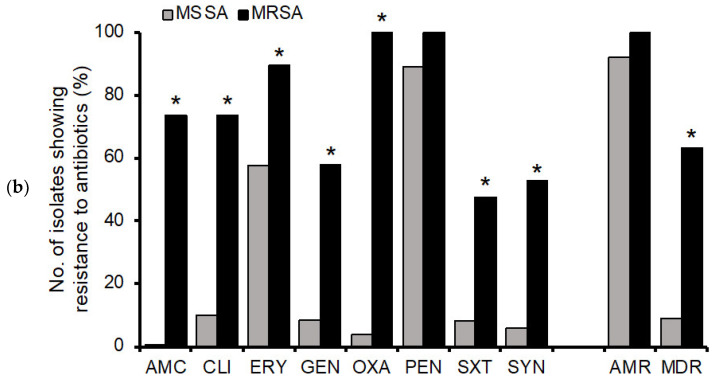
Multidrug resistance patterns of *S. aureus* isolated from wholesale and retail pork samples (**a**) and different groups, MSSA and MRSA (**b**), in Wuhan, China. AMC, amoxicillin/clavulanate; CLI, clindamycin; ERY, erythromycin; GEN, gentamicin; OXA, oxacillin; PEN, penicillin; SXT, trimethoprim/sulfamethoxazole; SYN, quinupristin/dalfopristin. AMR, resistant to at least one antimicrobial; MDR, resistant to three or more classes of antimicrobials. * Indicates *p* < 0.05.

The 518 *S. aureus* isolates were assessed for resistance to eight antimicrobial agents. Resistance to PEN was the most commonly observed resistance in the wholesale (89.0%) and retail 88.3%) pork isolates. Higher resistance rates for ERY were also noted in the isolates from wholesale (56.7%) and retail (60.5%) pork (Figure 1a; Supplementary Table S2). In addition, most isolates (92.7%) were resistant to at least one or more antimicrobial agents (Figure S1). The MDR isolates were detected in the isolates from wholesale (13.5%) and retail (5.6%) pork. Of the 518 *S. aureus* isolates, 499 (96.3%) were MSSA, and 19 (3.7%) were MRSA. Furthermore, the MRSA isolates showed significantly greater resistance to all eight antimicrobials tested in this study compared with the MSSA isolates (*p* < 0.05) (Figure 1b).

### 3.3. Multilocus Sequence Typing (MLST) of the S. aureus Isolates

The MLST results revealed that twenty-eight sequence types (STs) were identified in the *S. aureus* isolates from both wholesale and retail pork (Table 2). The predominant ST types was ST7 (57.5%), followed by ST5 (9.1%), ST3055 (4.3%), ST118 (3.7%), and ST9 (3.3%) in the isolates from wholesale and retail pork. Among them, ST7 (59.7%) was the most frequent genotype for the MSSA isolates (298/499), while ST9 (42.1%) was the most frequent genotype for the MRSA isolates (8/19). However, there was no significant difference in the rates of ST types between the wholesale and retail pork groups (data not shown).

### 3.4. Genotypic Characteristics of the S. aureus Isolates

**Figure 2:**
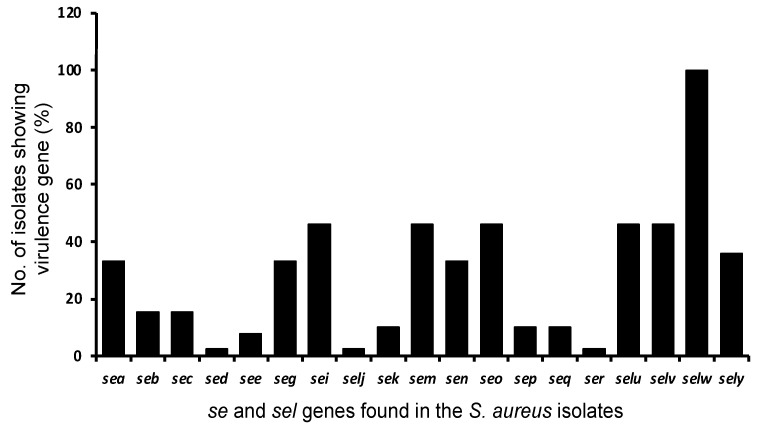
Distribution of different SE and SEl genes among the *S. aureus* isolates from wholesale and retail pork in Wuhan, central China.

We analyzed staphylococcal protein A gene (*spa*) profiles of the 39 representative *S. aureus* isolates from wholesale and retail pork based on the whole-genome sequences. The most commonly detected *spa* type in the MRSA isolates was t899, followed by t437, and the most detected SCC*mec* type was XII(9C2), followed by IVa(2B) (Table 3). In contrast, the most commonly detected *spa* type in the MSSA isolates was t091, followed by t6497. A total of 19 SEs and SEls genes were detected in the isolates, all of which carried *selw* (100.0%), followed by *sei*, *sem*, *seo*, *seu*, and *selv* (46.2%); *sey* (35.9%); and *sea*, *seg*, and *sen* (33.3%) (Figure 2). The gene spectrum of SE and SEl was relatively similar between the MRSA and MSSA strains (Table 3).

### 3.5. Whole-Genome Sequencing Analysis of S. aureus

**Figure 3:**
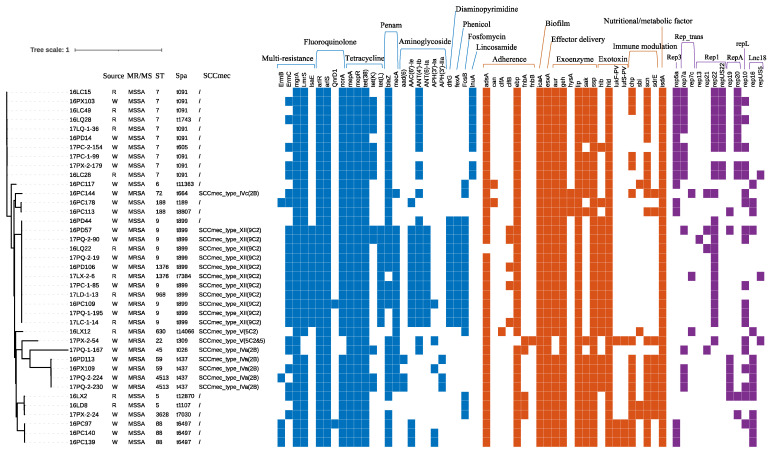
Core SNP tree of the 39 *S. aureus* isolates from wholesale and retail pork in Wuhan, China. All isolates came from wholesale markets (W) or retail markets (R). Different types of antimicrobial resistance genes, virulence genes, and plasmid replicators are displayed with the heat map (colored squares for positives), and the class of each gene is marked above the heat map (multiresistance: ErmB and ErmC: lincosamide, macrolide and streptogramin; mgrA: cephalosporin, fluoroquinolone, penam and peptide; tetracycline; LmrS: aminoglycoside, diaminopyrimidine, macrolide, oxazolidinone and phenicol; lsaE: lincosamide, macrolide, oxazolidinone, phenicol, pleuromutilin, streptogramin, and tetracycline). NCTC8325 was used as an *S. aureus* reference genome (NCBI genome: txid93061), and bootstrap values from 1000 replicates were performed but not shown on the branches.

After mapping with the reference genome (NCTC8325), a total of 151,721 variant sites were found in all 39 isolates, of which 92,265 core SNPs were extracted for the phylogenetic tree. The whole-genome assembly results showed that N50 was 101 to 694 kb, the number of contigs was 24 to 446, and the gene numbers were 2480 to 2872 Table S3. Based on the WGS, we further constructed the core genome SNP phylogeny of the 39 *S. aureus* isolates from wholesale and retail pork, as well as STs, Spa types, SCC*mec* types, antimicrobial resistance genes, virulence factor genes, and plasmids (Figure 3). The core SNP phylogenetic tree showed that ST9-t899-XII (9C2)-*selw* was the most frequent sequence type in MRSA (42.1%), while ST7-t091-*selw* (40.0%) was the most common type in MSSA. Isolates from different sources shared the same lineage with no significant differences in sampling time and market types. Multiple genes for different types of antimicrobials were analyzed, and the resistance genes for fluoroquinolone, tetracycline, aminoglycosides, and lincosamide were the most commonly detected; MRSA strains usually carried more resistance genes, such as *lsaE*, *tet(L)*, *AAC(6’)-Ie-APH(2’’)-Ia*, *ANT(6)-Ia*, *fexA*, *fosB*, and *dfrG*, suggesting a greater resistance level than that of MSSA. Twenty-five virulence genes were detected, and all isolates carried *icaA*, *isdA*, *lip*, *ssp*, *esxA*, *geh*, and *hld* (*n* = 39), followed by *adsA* (*n* = 38), *ebp* (*n* = 38), and *aur* (*n* = 37). A total of 12 plasmid types were detected, of which rep22 (59.0%) had the highest carrying rate, followed by rep10 (48.7%), rep5a (41.0%), rep7a (35.9%), rep16 (35.9%), rep20 (28.2%), repUS22 (23.1%), rep19 (18.0%), rep21 (10.3%), rep7c (5.1%), rep13 (5.1%), and repUS5c (5.1%). The proportion of strains carrying more than two plasmids was 87.2% (34/39). These results indicated that the isolates from wholesale and retail pork in central China carried multiple antimicrobial resistance and virulence genes that cause food poisoning and infectious diseases.

## 4. Discussion

In this study, we carried out PCR, MLST, and WGS to investigate the prevalence, antimicrobial resistance, SE and SEl, and genotypic characterization of *S. aureus* isolated from wholesale and retail pork in Wuhan, China. We provided comprehensive and detailed genotyping of multiple virulence factors, as well as publicly available data on *S. aureus* isolates from pork in China, including MRSA and MSSA. Our results showed that the prevalence (16.9%) of *S. aureus* in wholesale and retail pork was lower than that in previous studies, such as in Fujian and Guangdong (23.4%), Shanghai (28.1%), and other regions (47.7%) in China [19,32,43] and in North Dakota (49.3%) in the United States [44], but the prevalence was similar to that in Europe countries (15.0%) [22,45] and Iowa in the United States (18.2%) [46]. These results indicate that both wholesale and retail pork may serve as reservoirs of *S. aureus*, which may originate from *S. aureus*-positive animals, the surrounding environment, humans, and other sources during food processing and commercialization in meat counters at supermarkets and retail stores.

The prevalence of drug-resistant bacteria is increasing due to the excessive use of antibiotics. In this study, the prevalence (3.7%) of MRSA isolated from the wholesale and retail pork was similar to those in Europe countries (1.8–15.8%) [47], North American countries (1.9–9.6%) [48,49], and south China (6.3%) [32]. Notably, 92.3% of the *S. aureus* isolates from the pork revealed resistance to at least one tested antimicrobial, and 74.5% of the isolates exhibited an AMR pattern of ERY-PEN or PEN. We observed higher resistance rates to penicillin (88.8%) and erythromycin (58.1%) (Figure 1a), which have commonly been reported in *S. aureus* isolated from meat samples [29,44,48]. The higher rates of resistance may be related to the use of the antimicrobials to treat diseases and as feed additives or growth promoters in livestock [29]. The rate of MDR in MRSA isolates (63.2%) from wholesale and retail pork was significantly higher than that in MSSA isolates (9.0%; Figure 1b). Wholesale and retail pork contaminated with MDR *S. aureus* are potentially hazardous, and the food chain may be a key site for the transmission of resistance among livestock, the environment, and humans [29].

ST398 was first identified in pigs and swine workers but has since been found in other animals including cattle, poultry, and dogs as well as humans [15,16,17,18,39,45]. ST398 was the most dominant clone disseminating worldwide, especially in Europe, Asia, and North and South America (EFSA Panel on Animal Health and Welfare, 2022). In several countries, different human-adapted ST398 has been shown to circulate in the community and cause clinical MSSA infection in humans [21,27,50]. ST5, ST9, and ST15 were predominantly obtained from pigs and cattle in China and other Asian countries, indicating ST9 and ST398 might be endemic in animal-derived food production [14,29,32,51]. These results indicate that indirect transmission of *S. aureus* from livestock has resulted in clinical human infection, although the route of transmission in such cases has not been determined. In the present study, a total of 28 STs were identified in the 518 isolates from different pork sources. Of them, ST7 was the most predominant type in the isolates from wholesale and retail pork in China, which are different from the results of previous studies [16,29]. Notably, our results showed that ST7 was the most frequent type in MSSA isolates, while ST9 was the most common in MRSA isolates but not in MSSA. Recently, several studies have reported that ST9 is currently the most prevalent sequence type of LA-MRSA and a major endemic MRSA clone circulating in pigs in Asia countries [19,32,51]. These results indicate that pigs and pork could become important reservoirs for MRSA and increase the potential risk of human infections caused by LA-MRSA.

In the present study, a variety of virulence factors were detected from strains isolated from pork, which are important toxins causing food poisoning and infection in humans and animals. A total of 19 SEs and SEls genes were detected in the isolates from the pork, and *selw* (100.0%), a newly reported SE-like toxin, was the most common toxin type, followed by *sei*, *sem*, *seo*, *seu*, and *selv* (46.2%); *sey* (35.9%); and *sea*, *seg*, and *sen* (33.3%). These results are significantly different from previous reports of pork from other regions of China [14,28,29,32]. Combined with the genotypes detected in this study, ST7-t091-*selw* was the most common genotype profile (40.0%) among MSSA isolates from wholesale and retail pork, which was the first report that MSSA ST7-t091-*selw* is the main lineage in pork in China. On the other hand, for MRSA isolated from the wholesale and retail pork, ST9-t899-XII (9C2)-*selw* was the predominant genotype profile (42.1%), which was similar to previous studies [17,52]. Importantly, the *S. aureus* isolated from pork carried multiple antimicrobial resistance and virulence genes, especially many types of SE and SEI toxin genes, which are the important causative toxins inducing food poisoning and infectious diseases in humans [53,54]. These results demonstrate that strains isolated from pork may have interspecies transmission of adaptability and virulence in different hosts and foods [3,52].

In conclusion, our findings indicated that the examined wholesale and retail pork were generally contaminated with *S. aureus*. ST7-t091-*selw* was the most predominant genotype profile in MSSA isolates, while ST9-t899-XII(9C2)-*selw* was the most common genotype profile in MRSA isolates from pork in Wuhan, China. In addition, the isolates exhibited multiple antimicrobial resistances and possessed many types of virulence factors, including classic SE types and newly reported SE and SEl types. Our data indicate that multiple virulence genes detected in the *S. aureus* isolates, especially the newly reported SE and SEl genes, reveal new lurking threats to food safety and public health from wholesale and retail pork. These results demonstrate that good hygiene and good production practices to prevent interspecies transmission and cross-contamination of *S. aureus* in the pig–pork chain are of great significance to public health.

## Figures and Tables

**Table 1:** Overview of *S. aureus* prevalence in wholesale and retail pork in Wuhan, central China.

Pork Source (Markets)	Season	No. of Samples	*S. aureus*				MRSA		MSSA
			No. of Isolates	Prevalence (%)	95% CI * (%)	No. of Isolates	Prevalence (%)	No. of Isolates	Prevalence (%)
Wholesale	Spring	576	85	14.8 ^β,γ^	12.0–17.9	3	3.5	82	96.5
	Summer	594	71	12.0 ^γ^	9.5–14.8	2	2.8	69	97.2
	Autumn	661	106	16.0 ^β^	13.3–19.1	6	5.7	100	94.3
	Winter	522	94	18.0 ^α,β^	14.8–21.6	3	3.2	91	96.8
	Total	2353	356	15.1	13.7–16.6	14	3.9	342	96.1
Retail	Spring	153	37	24.2 ^b^	17.6–31.8	1	2.7	36	97.3
	Summer	194	31	16.0 ^b^	11.1–21.9	1	3.2	30	96.8
	Autumn	186	32	17.2 ^b^	12.1–23.4	2	6.3	30	93.8
	Winter	181	62	34.3 ^a^	27.4–41.7	1	1.6	61	98.4
	Total	714	162	22.7	19.7–25.9	5	3.1	157	96.9
Total		3067	518	16.9	15.6–18.3	19	3.7	499	96.3

Numbers with different letters (α, β, and γ, or a and b) indicate significant difference among seasons, and numbers sharing the same letter indicate no significant difference. * CI: confidence interval.

**Table 2:** Overview of *S. aureus* prevalence in wholesale and retail pork in Wuhan, central China.

MLST	No. of *S. aureus*		No. from Markets		No. of MRSA		No. of MSSA	
Type	(*n* = 518, %)		Wholesale	Retail	(*n* = 19, %)		(*n* = 499, %)	
ST7	298	57.5	200	98	-	-	298	59.7
ST5	46	9.1	22	24	-	-	46	9.2
ST3055	22	4.3	20	2	-	-	22	4.4
ST188	19	3.7	15	4	-	-	19	3.8
ST9	17	3.3	14	3	8	42.1	9	1.8
ST15	14	2.7	11	3	-	-	14	2.8
ST25	14	2.7	14	-	-	-	14	2.8
ST1	10	1.9	6	4	-	-	10	2
ST6	10	1.9	8	2	-	-	10	2
ST88	10	1.9	10	-	-	-	10	2
ST1640	10	1.9	-	10	-	-	10	2
ST72	7	1.4	3	4	1	5.3	6	1.2
ST2250	6	1.2	5	1	-	-	6	1.2
ST630	5	1	1	4	1	5.3	4	0.8
ST1281	5	1	5	-	-	-	5	1
ST59	4	0.8	4	-	2	10.5	2	0.4
ST398	4	0.8	4	-	-	-	4	0.8
ST672	3	0.6	2	1	-	-	3	0.6
ST338	2	0.4	2	-	-	-	2	0.4
ST1376	2	0.4	1	1	2	10.5	-	-
ST1920	2	0.4	2	-	-	-	2	0.4
ST4513	2	0.4	2	-	2	10.5	-	-
ST22	1	0.2	1	-	1	5.3	-	-
ST45	1	0.2	1	-	1	5.3	-	-
ST968	1	0.2	1	-	1	5.3	-	-
ST1821	1	0.2	-	1	-	-	1	0.2
ST1921	1	0.2	1	-	-	-	1	0.2
ST2315	1	0.2	1	-	-	-	1	0.2

**Table 3:** ST, *spa*, SCC*mec*, and SE or SEl genes identified in the *S. aureus* isolates from wholesale and retail pork in Wuhan, central China.

*S. aureus*	MLST	Spa	*SCCmec*	SE or SEl Genes
MRSA	ST9	t899	XII(9C2)	*sea*, *selw*
		t899	XII(9C2)	*sea*, *selw*
		t899	XII(9C2)	*seb*, *sec*, *seg*, *sei*, *sem*, *sen*, *seo*, *selu*, *selv*, *selw*
		t899	XII(9C2)	*seg*, *sei*, *sem*, *sen*, *seo*, *selu*, *selv*, *selw*
		t899	XII(9C2)	*sei*, *sem*, *seo*, *selu*, *selv*, *selw*, *sey*
		t899	XII(9C2)	*seb*, *sec*, *selw*
		t899	XII(9C2)	*sea*, *sec*, *sek*, *sep*, *seq*, *selw*, *sey*
		t899	XII(9C2)	*sea*, *selw*
	ST22	t309	V(5C2&5)	*seg*, *sei*, *sem*, *sen*, *seo*, *selu*, *selv*, *selw*, *sey*
	ST45	t026	IVa(2B)	*sea*, *selw*
	ST59	t437	IVa(2B)	*sei*, *sem*, *seo*, *selu*, *selv*, *selw*
		t437	IVa(2B)	*sea*, *selw*
	ST72	t664	IVc(2B)	*sei*, *sem*, *seo*, *selu*, *selv*, *selw*, *sey*
	ST630	t14066	V(5C2)	*seb*, *sec*, *sek*, *sep*, *seq*, *selw*, *sey*
	ST968	t899	XII(9C2)	*seg*, *sei*, *sem*, *sen*, *seo*, *selu*, *selv*, *selw*, *sey*
	ST1376	t899	XII(9C2)	*sea*, *selw*
		t7384	XII(9C2)	*selw*
	ST4513	t437	IVa(2B)	*sea*, *selw*
		t437	IVa(2B)	*sea*, *selw*
MSSA	ST5	t12870	-	*seg*, *sei*, *sem*, *sen*, *seo*, *selu*, *selv*, *selw*
		t1107	-	*sea*, see, *sek*, *sep*, *seq*, *selw*
	ST6	t11363	-	*sei*, *sem*, *seo*, *selu*, *selv*, *selw*
	ST7	t065	-	*seg*, *sei*, *sem*, *sen*, *seo*, *selu*, *selv*, *selw*
		t091	-	*sea*, *selw*
		t091	-	*seg*, *sei*, *sem*, *sen*, *seo*, *selu*, *selv*, *selw*, *sly*
		t091	-	*sea*, *see*, *selw*
		t091	-	*sea*, *seb*, *sec*, *sed*, *see*, *seg*, *sei*, *selj*, *sem*, *sen*, *seo*, ser, *selu*, *selv*, *selw*
		t091	-	*sei*, *sem*, *seo*, *selu*, *selv*, *selw*, *sey*
		t091	-	*seg*, *sei*, *sem*, *sen*, *seo*, *selu*, *selv*, *selw*
		t091	-	*selw*
		t091	-	*selw*
		t1743	-	*seb*, *sec*, *sek*, *sep*, *seq*, *selw*, *sey*
	ST9	t899	-	*sea*, *selw*
	ST88	t6497	-	*selw*
		t6497	-	*seg*, *sei*, *sem*, *sen*, *seo*, *selu*, *selv*, *selw*, *sey*
		t6497	-	*seg*, *sei*, *sem*, *sen*, *seo*, *selu*, *selv*, *selw*, *sey*
	ST188	t189	-	*seg*, *sei*, *sem*, *sen*, *seo*, *selu*, *selv*, *selw*, *sey*
		t8807	-	*seg*, *sei*, *sem*, *sen*, *seo*, *selu*, *selv*, *selw*, *sey*
	ST3628	t7030	-	*selw*

## Data Availability

Data is contained within the article or Supplementary Material.

## Supplementary Materials

The following supporting information can be downloaded at: https://www.mdpi.com/article/10.3390/foods11244114/s1, Figure S1: Correlation between high/low MIC values and AMR gene profiles, Table S1: MIC breakpoints of S. aureus in this study, Table S2: All MICs values of 518 isolates from wholesale and rerail pork in Wuhan, central Chiana, Table S3: The whole genome assembly results.
